# Boosting the Electrolysis of Monosaccharide-Based Streams in an Anion-Exchange Membrane Cell

**DOI:** 10.1021/acs.energyfuels.4c00136

**Published:** 2024-05-20

**Authors:** J. Serrano-Jiménez, A.R. de la Osa, P. Sánchez, A. Romero, A. de Lucas-Consuegra

**Affiliations:** † Department of Chemical Engineering, School of Chemical Sciences and Technologies, University of Castilla-La Mancha, Avda. Camilo José Cela 12, E-13071 Ciudad Real, Spain; ‡ Department of Chemical Engineering, Higher Technical School of Agronomical Engineers, University of Castilla-La Mancha, Ronda de Calatrava 7, E-13071 Ciudad Real, Spain

## Abstract

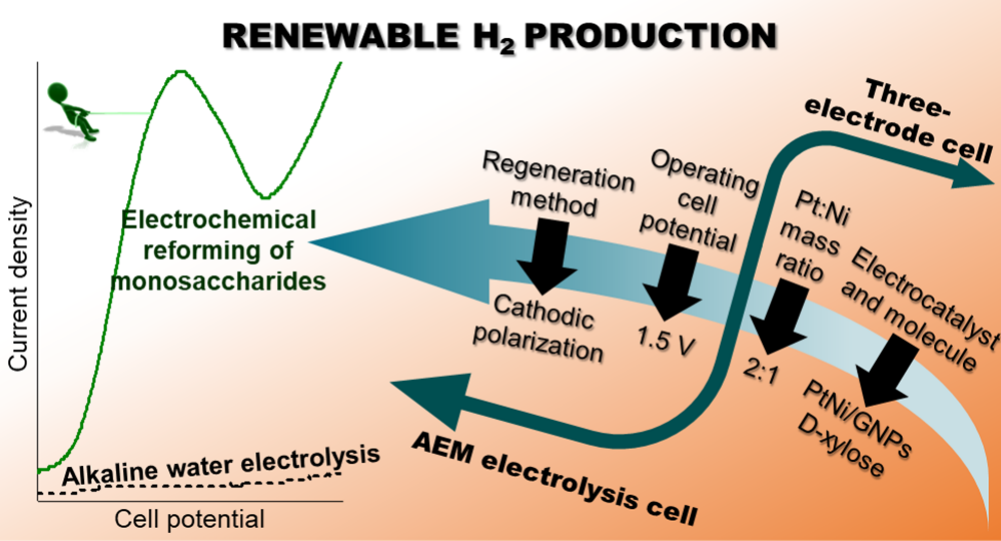

A systematic study on the electrochemical reforming of monosaccharides (fructose, glucose, and xylose) using Pt-based anodic electrocatalysts is here presented for the first time to completely optimize the anodic catalyst and electrolyzer operating conditions. First, the electro-oxidation of each molecule was studied using a monometallic (Pt) and two bimetallic (PtNi and PtCo) anodic electrocatalysts supported on graphene nanoplatelets (GNPs). Tests in a three-electrode cell showed superior electrochemical activity and durability of PtNi/GNPs, especially at potentials higher than 1.2 V vs RHE, with the highest electrocatalytic activity in d-xylose electro-oxidation. Then, monometallic (Pt and Ni) and bimetallic electrocatalysts with different Pt:Ni mass ratios (1:1 and 2:1) were studied for d-xylose electro-oxidation, with the 2:1 mass ratio presenting the best results. This electrocatalyst was selected as the most suitable for scale-up to an anion-exchange membrane electrolyzer, where the optimal operating potential was determined. Additionally, stable operating conditions of the electrolyzer were achieved by cyclic H_2_ production and cathodic regeneration polarization steps. This led to suitable and reproducible H_2_ production rates throughout the production cycles for renewable hydrogen production from biomass-derived streams.

## 1 Introduction

The search for renewable fuels has been motivated as a response to address both the emission of polluting gases and the depletion of fossil fuels. Accordingly, hydrogen produced from electrochemical processes powered by renewable energies (green hydrogen) represents a promising approach to energy storage. In this sense, the lower potential required in the electrochemical reforming of biomass-based molecules, such as carbohydrates or biopolymers, emerges as a cost-saving alternative to water electrolysis.^1,2^ Within carbohydrates, monosaccharides are widely present in natural plants. However, since many of them are used to feed mankind, this creates a conflict between fuel production and food chain.^3^ However, they can also be obtained from the hydrolysis of complex carbohydrate molecules present in non-edible biomass (such as hemicellulose or lignocellulose) through enzymes or highly concentrated solutions of acids or bases.^4−6^ In this context, glucose and xylose are the most abundant hydrolysates derived from lignocellulosic biomass, whereas fructose is also produced to a lesser extent.^7,8^ Additionally, glucose and fructose are also present in winery wastewater in elevated concentrations in the form of unfermented sugars.^9^

Chemical oxidation of monosaccharides has been extensively used to produce biosourced value-added chemicals, such as xylonic or gluconic acids (from xylose and glucose oxidation, respectively), which can be used in a wide variety of applications, including the synthesis of various biodegradable chemicals or the production of food and drugs.^10,11^ However, these processes involve the use of environmentally hazardous compounds. Therefore, the electrochemical reforming of monosaccharides is proposed as a cleaner alternative. In a typical anion-exchange membrane (AEM) electrolyzer, aqueous solutions of monosaccharides are fed into the anodic compartment and oxidized into value-added platform molecules, whereas highly pure hydrogen (99.999% purity) is obtained from the cathodic compartment. These organic compounds can also be used as sustainable fine chemicals, hence empowering a circular economy model based on biorefineries.

Alkaline electro-oxidation of glucose and xylose (fructose to a lesser extent) has been reported using Pt-based anodic electrocatalysts due to their exceptional electrochemical activity.^12,13^ Glucose electro-oxidation has also been explored in medical applications, such as blood sensors for diabetes monitoring.^14^ However, due to the low resistance of Pt to the poisoning effect of intermediate reaction species, the addition of transition metals (such as Ni or Co) in bimetallic electrocatalysts may improve both the electrochemical activity and durability, as well as reduce the amount of Pt used.^15^ Additionally, Ni-based electrocatalysts are widely studied in alkaline media, especially in water electrolysis.^16^ However, few publications can be found concerning alkaline glucose electro-oxidation with PtNi or PtCo electrocatalysts. Additionally, the electro-oxidation of xylose and fructose has not yet been sufficiently explored in the literature, especially using these bimetallic electrocatalysts.

Considering this, the first part of this work involves a systematic study of the electro-oxidation of d-glucose, d-xylose, and d-fructose using monometallic (Pt) and bimetallic (PtNi and PtCo) electrocatalysts supported on graphene nanoplatelets (GNPs). The selection of this carbonaceous support was based on a previous study from our group, where the use of GNPs in a Pt (20 wt %) anodic catalyst led to superior activity and stability in ethanol electro-oxidation in comparison to when the extensive Vulcan XC-72 was used. This was ascribed to a higher BET surface area and a higher content of nitrogen (which may act as doping agent) than the other supports.^17^ Then, the most active and stable bimetallic electrocatalyst was selected to optimize the Pt:M mass ratio to enhance the electro-oxidation of the monosaccharide, which provided the best results. Finally, the optimized electrocatalyst was scaled-up to an AEM electrolyzer, where the cell operating potential in the AEM cell was optimized. Moreover, a new procedure for anode regeneration alternative to open-circuit potential (OCP) was studied, achieving a higher stability and hence, a practically constant hydrogen production.

In this context, this work offers advances and new insights into potential industrial application prospects in the field of green hydrogen production via biomass-assisted electrolysis.

## 2 Experimental Section

### 2.1 Materials

GNPs used as the anodic catalyst support were purchased from Nanografi Nano Technology (purity 99.9%, particle size 1.5 μm). Commercial Pt (20 wt %)/C catalyst used as cathode was supplied by Alfa Aesar. Hexachloroplatinic acid (H_2_PtCl_6_·*x*H_2_O), cobalt(II) chloride hexahydrate (CoCl_2_·6H_2_O), Nafion 5% wt. solution, and ethylene glycol (99.8% purity) were acquired from Sigma-Aldrich. d-(+)-glucose, d-(+)-xylose, and d-(−)-fructose were provided by Thermo Fisher Scientific, Sigma-Aldrich, and Panreac, respectively. Nickel(II) chloride hexahydrate (NiCl_2_·6H_2_O) and sodium hydroxide (NaOH) were purchased from Panreac. 2-Propanol was supplied by Honeywell. Deionized water was produced in the laboratory.

### 2.2 Synthesis of Anodic Electrocatalysts

Monometallic and bimetallic anodic electrocatalysts were synthesized using the modified polyol method, which was previously demonstrated in our group to be suitable for obtaining small crystallite sizes in PtNi and PtCo electrocatalysts.^17,18^ A metal loading of 40 wt % was established for all electrocatalysts, which was demonstrated to be optimal in terms of electrochemical activity and durability on GNPs, according to a previous work from our group involving the electrochemical reforming of ethanol.^17^

First, a certain amount of the precursor salts (H_2_PtCl_6_·H_2_O and NiCl_2_·6H_2_O or CoCl_2_·6H_2_O) and 400 mg of NaOH were added to 50 mL of ethylene glycol to obtain a NaOH concentration of 0.2 M. This mixture was stirred at 500 rpm for 1 h to dissolve the solids and subsequently heated to 190 °C for 2 h to complete the reduction of precursor salts into metals. Then, the GNPs support was added to the hot solution and left to cool down to room temperature under stirring for 48 h, hence allowing the metal particles to settle on the support. After this step, the electrocatalyst was washed with 1.2 L of deionized water, filtered, and dried at room temperature overnight. Then, the electrocatalyst was dried in an oven at 100 °C for 6 h until completely dry.

### 2.3 Preparation of Catalytic Inks and Electrodes

Catalytic inks were prepared according to previous studies of our group.^17,19,20^ In the case of inks for three-electrode cell testing, 2 mg of the anodic catalyst, 8 μL of Nafion 5 wt % ionomer solution, 250 μL of deionized water, and 750 μL of 2-propanol were mixed and ultrasonicated (110 W/50–60 Hz, Selecta) for 3 h. Inks were continuously stirred to maintain a homogeneous suspension. Inks used in the AEM electrolyzer were prepared by mixing a certain amount of the cathodic or the synthesized anodic electrocatalysts with the ionomer solution (ionomer solution/catalyst mass ratio of 3.64) and enough of 2-propanol. Then, the inks were kept under continuous stirring to completely disperse the catalyst powder. Once dispersed, the electrocatalysts were deposited on a gas diffusion layer (GDL) by airbrushing (spray coating method) in a catalyst-coated substrate (CCS) configuration. A metal loading of 1.5 mg_metal_·cm^–2^ for the anode was established based on a previous work of our group,^17^ whereas a metal loading of 0.5 mg_metal_·cm^–2^ was established for the cathode.

### 2.4 Physicochemical Characterization

The crystalline structures present in both the GNPs support and the anodic electrocatalysts were determined by X-ray diffraction (XRD) using a Philips PW-1700 equipment with a nickel-filtered Cu Ka (λ = 1.5418 Å) in the range of 10° < 2θ < 90° and a step size of 0.02°·step^–1^ and a scanning speed of 2 s·step^–1^. Detected species were identified according to the Joint Committee on Powder Diffraction Standards (JCPDS).

The average and distribution of crystallite sizes of Pt, PtNi, and PtCo electrocatalysts were determined by high-resolution transmission electron microscopy (HRTEM) using a FEI Talos F200X microscope operating at an accelerating voltage of 200 kV.

The textural properties of the support and the anodic electrocatalysts were evaluated in a Micromeritics ASAP 2010 sorptometer using N_2_ at 77 K as an adsorbate. The degassing method consisted of applying a light vacuum at 50 °C for 1 h, followed by a gross vacuum at 200 °C and for 4 h. The surface area was determined according to the Brunauer–Emmett–Teller (BET) method. The mesoporous and microporous character of the materials was determined according to the Barrett–Joyner–Halenda (from the desorption isotherm) and Horvath–Kawazoe methods, respectively.

### 2.5 Electrochemical Experiments in a Three-Electrode Electrochemical Cell

Electrochemical tests were carried out in a glass three-electrode cell using a glassy carbon electrode (GCE) with a geometric area of 0.07 cm^2^ as a working electrode, a platinum foil (1 cm^2^) connected to a Pt wire as a counter electrode, and a Ag/AgCl (3 M KCl) reference electrode equipped with a double junction and a ceramic diaphragm. All electrodes were acquired from Metrohm. Electrochemical experiments were conducted using an Autolab PGSTAT302N potentiostat-galvanostat, controlled by the NOVA 1.6.013.0 software. GCE was polished with an alumina paste (grain size 0.3 μm) for 1 min and washed with enough deionized water before ink deposition. Then, 5.5 μL of catalytic ink (60 μg_metal_·cm^–2^) was deposited on the GCE and allowed to dry at room temperature.

Cyclic voltammetry (CV) experiments were carried out in a 0.5 M NaOH solution (previously purged with ultrapure N_2_ for 15 min) between −0.15 and 1.4 V vs RHE (reversible hydrogen electrode) at a scan rate of 50 mV·s^–1^ for five cycles, until stabilization. Thus, the displayed CV curves correspond to the fifth cycle. Afterward, the electro-oxidation of the d-monosaccharides under study (fructose, glucose, and xylose) was explored using a monosaccharide concentration of 0.1 and 0.5 M NaOH. All aqueous solutions were prepared right before testing to avoid as much as possible the self-reaction between NaOH and the monosaccharide.^21,22^ Thereby, the solutions did not show a significant degradation over the experimental time. CV tests were carried out in a potential range from 0.2 to 1.5 V vs RHE at a scan of 10 mV·s^–1^ for five cycles. In the case of chronoamperometry (CA) experiments, a potential of 1.1 V vs RHE was applied for three cycles of 15 min each. All potentials indicated in the results section are referred to vs RHE.

### 2.6 Electrochemical Reforming in an AEM Electrolyzer

The electrochemical reforming tests were performed in an AEM electrolyzer with an electrode area of 4 cm^2^. Carbon paper (TGP-H-90 from Fuels Cells Earth, 280 μm thickness) was used as the GDL and for the preparation of the CCS configuration electrodes, which were placed at both sides of the AEM membrane (Sustainion X37-50 grade T from Dioxide Materials, 50 μm thickness), forming the membrane electrode assembly (MEA). The MEA was in contact with two grade 1 titanium bipolar plates with a thickness of 2 mm and a groove volume of 0.25 cm^3^. Two Teflon gaskets were incorporated to avoid a short circuit between the bipolar plates. All these elements were confined between two aluminum plates (1 cm thickness) tightened with M5 Allen bolts at 3 N·m.

All of the electrochemical experiments were performed at 50 °C using an analogic controller equipped with a thermocouple wire and two electric resistors inserted in the steel plates. To avoid membrane dehydration, a 0.5 M NaOH solution was fed to the cathodic compartment at a flow rate of 2.5 mL·min^–1^, whereas the solution for the anode was fed at 1.5 mL·min^–1^ using a multichannel peristaltic pump (Heidolph C4) for this purpose. A Pt (20 wt %)/C was used as a cathode, whereas the previously optimized bimetallic electrocatalyst was used as the anode. The electrochemical measurements were conducted with a potentiostat/galvanostat Vertex.10A from Ivium Technologies, controlled by IviumSoft software. Linear sweep voltammetry (LSV) tests were performed in a cell potential range from 0 to 1.5 V at a scan rate of 20 mV·s^–1^. Chronopotentiometry (CP) experiments were performed at 12.5, 25, and 37.5 mA·cm^–2^ (0.05, 0.10, and 0.15 A) for 15 min each. CA tests were first conducted at the cell potentials of 0.7, 1.1, and 1.5 V for 15 min, intercalating OCP regeneration cycles of 1 min between the operating cycles. Then, another regeneration method was explored, consisting of a cathodic polarization at a cell potential of −1.5 V for 1 min between the operating cycles at 1.5 V. The products formed in the electrochemical reforming process were analyzed by Fourier transform infrared (FTIR) spectroscopy in PerkinElmer equipment equipped with the universal attenuated total reflectance accessory in the range of 500–4000 cm^–1^ with a resolution of 8 cm^–1^.

## 3 Results and Discussion

### 3.1 Study of the Synthesized Anodic Electrocatalysts

The first part of this work focuses on the electro-oxidation of the studied monosaccharides using monometallic (Pt/GNPs) and bimetallic (PtNi/GNPs and PtCo/GNPs) electrocatalysts with a metal loading of 40 wt % and a Pt:M mass ratio of 2:1 (for the bimetallic ones). The results from both physicochemical and electrochemical characterizations are shown below.

#### 3.1.1 Physicochemical Characterization

**Figure 1:**
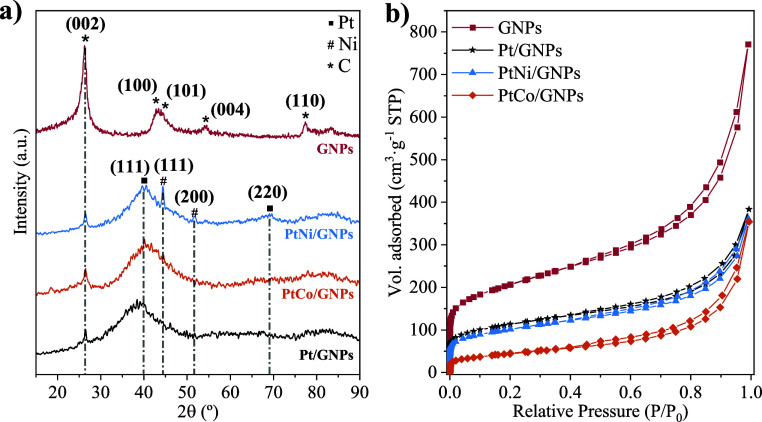
Physicochemical characterization: (a) XRD patterns and (b) N_2_ adsorption–desorption isotherms of the Pt/GNPs, PtNi/GNPs, and PtCo/GNPs electrocatalysts and the GNPs support.

Electrocatalysts were characterized by XRD, N_2_ adsorption–desorption, and HRTEM techniques. Figure 1a displays the XRD patterns of the GNPs support and anodic electrocatalysts. The GNPs support showed a sharp peak at a diffraction angle of 2θ = 26.26° corresponding to the (002) reflection of hexagonal graphitic structures, hence demonstrating the highly crystalline morphology of this material.^23,24^ This peak was also observed in the electrocatalysts to a lesser extent. Other minor peaks at diffraction angles of 42.78°, 43.94°, 54.14°, and 77.38° were also observed in the GNPs pattern, which were attributed to (100), (101), (004), and (110) reflections of graphitic structures (JCPDS no. 00-001-0646).^25−27^ The Pt/GNPs electrocatalyst also presented a broad peak centered at 2θ = 39.10°. This peak can be indexed mainly to the reflection of the (111) plane, with a minor overlapping contribution of the (200) plane. These reflections are commonly associated with the face-centered cubic (fcc) structures of Pt (JCPDS no. 00-001-1190).^28^ The shift toward higher 2θ values in the case of bimetallic electrocatalysts indicates a decrease in the lattice parameter compared to that of Pt, indicating a higher degree of alloying. This suggests partial integration of Ni or Co into the Pt crystalline structure. Table S1 shows the alloy degrees, which were calculated according to the Vegard’s law.^29^ Thus, PtCo/GNPs electrocatalyst exhibited the highest alloy degree (30.7%), almost double that provided by PtNi/GNPs. The peak corresponding to the reflection of the Pt (220) plane (2θ = 67.6°) was slightly visible for PtNi/GNPs and almost practically negligible for Pt/GNPs and PtCo/GNPs. This peak is typically used to estimate the average particle size according to the Debye–Scherrer equation. However, no noticeable peaks of this reflection were clearly distinguished, hence suggesting an average particle size lower than the detection limit of the diffractometer (4 nm).^30^ An intense peak at 2θ = 44.46° and a weak peak at 2θ = 51.82° were observed only for the PtNi/GNPs electrocatalyst, which are indexed to (111) and (200) reflections of reduced Ni (JCPDS no. 00–004–0850).^31^ Note that no significant peak was appreciated at 2θ ≈ 62.88° in this case, typically found for NiO (220) reflection.^31,32^ In the case of the PtCo/GNPs electrocatalyst, the peaks typically associated with the Co structures at diffraction angles of 44°, 52°, and 76°, which correspond to (111), (200), and (220) planes of Co structures (JCPDS No. 01-089-4307), were complicated to distinguish due to the overlap of these signals with those of Pt planes.^18^

In order to provide further information concerning the crystallite size of the electrocatalysts, HRTEM characterization was performed. Figure S1 (Supporting Information) presents the HRTEM images and the corresponding crystallite size distribution, considering around 900 crystallites. All of the data obtained were fitted to a normal distribution. A better homogenization of the crystallites can be observed in the case of Pt/GNPs and PtNi/GNPs, with a small formation of crystallite clusters. A narrower distribution was observed in both cases, with average crystallite sizes of 2.4 ± 0.7 and 2.6 ± 0.6 nm for Pt/GNPs and PtNi/GNPs, respectively. Thus, sizes between 1.5 and 3 nm entail around 80% of the counted crystallites in both cases. The PtCo/GNPs electrocatalyst presented an apparently lower level of crystallite homogenization, which led to a wider size distribution. Accordingly, around 80% of the crystallites presented a size ranging from 2 to 3.5 nm. In addition, the presence of crystallites larger than 4.5 nm is more perceptible, obtaining an average size of 3.0 ± 1.2 nm.

The textural properties of both the GNP support and the prepared electrocatalysts were explored by N_2_ adsorption/desorption. The corresponding isotherms are shown in Figure 1b, whereas the data extracted from the experiments are collected in Table S2. All isotherms can be categorized as type I–IV according to the IUPAC classification, characteristic of mesoporous materials with a moderate microporous contribution.^33^ Thus, the first region at low relative pressures (close to 0) is distinctive of microporous materials with relatively small external surfaces. A moderate increase in the N_2_ adsorbed volume was appreciated at relative pressures between 0.05 and 0.7, characteristic of mesoporous materials in which a multilayer adsorption of N_2_ occurs. A characteristic H3-type hysteresis loop was observed from a relative pressure of ∼0.4, associated with the capillary condensation phenomenon formed in mesopores.^33^ Finally, a steep growth of the adsorbed volume was noted for relative pressures above 0.8, attributed to the presence of larger pores.^18^ Although the isotherms showed similar trends for all materials, a lower N_2_ adsorption level was reported for the metal electrocatalysts compared to GNPs, which agrees with their corresponding surface areas and pore volumes (Table S2). It can be deduced that with the incorporation of the metal (Pt, PtNi, or PtCo), the BET surface area of the electrocatalyst decreases significantly since the metal particles are deposited in both micro- and mesopores of GNPs. This causes a slight pore blockage, which is also supported by the simultaneous decrease of meso- and micropore volumes. On the other hand, a slightly lower meso- and micropore volume (and hence, total pore volume) of the PtNi/GNPs electrocatalysts was observed compared to Pt/GNPs, with PtCo/GNPs exhibiting the lowest micro- and mesopore volumes and thus the lowest BET surface area.

#### 3.1.2 Electrochemical Experiments in a Three-Electrode Cell

##### 3.1.2.1 Cyclic Voltammetry Tests

The electrochemical behavior of the three anodic electrocatalysts was determined by cyclic voltammetry (CV) experiments in 0.5 M NaOH (Figure S2). Displayed CV curves exhibit typical profiles found in the literature for electrocatalysts with polycrystalline Pt, showing the characteristic regions of hydrogen underpotential deposition or HUPD between −0.15 and 0.5 V and double layer (0.5–0.6 V). At potentials above 0.6 V, the formation of metallic oxides is favored, which are reduced during the backward scan.^30,34^ Regarding the HUPD region, various peaks can be found which correspond to processes associated with hydrogen adsorption/desorption and the subsequent hydrogen evolution and hydrogen oxidation reactions, respectively). Moreover, the simultaneous adsorption/desorption of OH species onto various sites of the Pt surface occurs.^34−39^ Considering this, a first peak at 0.18 V, which is commonly associated with the activity of (110)-type sites of Pt^36^ resulted to be higher in the case of Pt/GNPs in comparison to PtNi/GNPs, indicating a preferential orientation toward these sites in the monometallic catalyst. Two smaller peaks (centered at 0.35 and 0.4 V) were also observed, which are associated with (100)-type sites,^40,41^ showing a similar intensity in these electrocatalysts. However, all of these peaks were observed to a lesser extent in PtCo/GNPs, probably attributed to its elevated alloying degree, which could result in variations in the Pt crystal orientation or even partial coverage of Pt monocrystal surfaces by Co structures. This effect may foster an inhibition of the H/OH adsorption–desorption process, suggesting a poorer electrochemical activity toward the electro-oxidation process.^18,42^

After that, the electro-oxidation of three monosaccharides (d-fructose, d-glucose, and d-xylose) was explored using the synthesized Pt/GNPs, PtNi/GNPs, and PtCo/GNPs electrocatalysts.

##### 3.1.2.2 Electrochemical Experiments in d-Glucose

**Figure 2:**
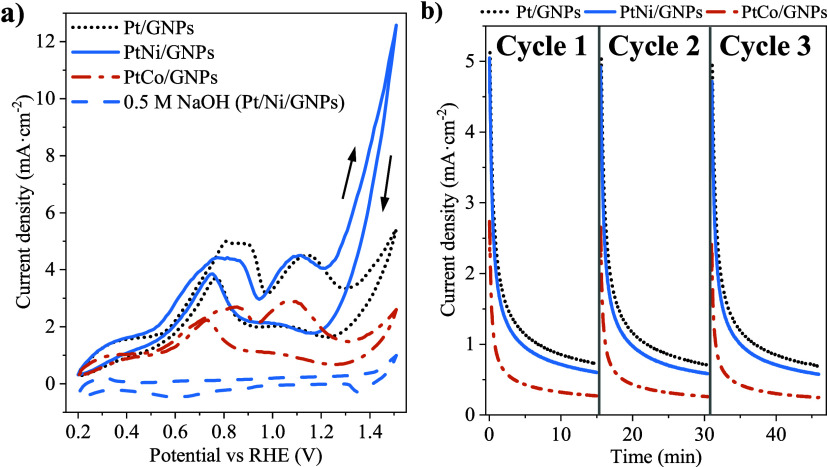
Electrochemical characterization of mono and bimetallic electrocatalysts in 0.1 M d-glucose + 0.5 M NaOH or 0.5 M NaOH (blank) at room pressure and temperature: (a) CV experiments at 10 mV·s^–1^ and (b) CA tests at 1.1 V vs RHE.

First, the influence of the NaOH concentration was explored in the d-glucose electro-oxidation. Figure S3 displays the CVs obtained in 0.1 M d-glucose varying the NaOH concentration between 0.1 and 1 M using PtNi/GNPs. As expected, the poorest activity was achieved with 0.1 M NaOH, probably due to the lower ionic conductivity of the liquid electrolyte. A considerable increase in the current density was observed with 0.5 M NaOH, especially at more elevated potentials, whereas only slightly higher current density values were reached by doubling the NaOH concentration (1 M). However, a higher concentration may favor the chemical reaction between NaOH and d-glucose into a wide variety of products.^13^ For this reason, 0.5 M NaOH was set as the optimal concentration in terms of electrochemical activity and the amount of NaOH used to conduct the following experiments. After this, the electrochemical behavior of Pt/GNPs, PtNi/GNPs, and PtCo/GNPs was explored in d-glucose electro-oxidation. Figure 2 shows the experimental information extracted from the CV and CA tests. For comparison purposes, a blank CV test was carried out in 0.5 M NaOH using PtNi/GNPs. It can be observed that an evident poorer activity was achieved with 0.5 M NaOH which improves when d-glucose is added due to the relatively slower kinetics of the oxygen evolution reaction of water electrolysis with respect to the electro-oxidation of glucose.^20,30^ Thus, all of the electrocatalysts presented a peak between 0.8 and 0.9 V and another centered at 1.1 V during the forward scan, whereas a peak at ∼0.75 V was observed in the backward scan. The first peak could be ascribed to a first oxidation in form of dehydrogenation of the anomeric group of the d-glucose molecule into various products on the Pt surface, while the second one could be attributed to further oxidation of the intermediate reaction products formed at lower potentials, simultaneously with fresh d-glucose molecules.^12,43^ Thus, Pt/GNPs and PtNi/GNPs exhibited similar current density values at 1.1 V versus RHE (∼4.5 mA·cm^–2^), showing Pt/GNPs a slightly higher value in the first peak (5 mA·cm^–2^ at 0.8 V). These results are competitive to those reported in the literature concerning glucose electro-oxidation in alkaline media, as listed in Table S3. Based on this, a noticeable difference was observed at higher potentials, reaching PtNi/GNPs with the highest current density and PtCo/GNPs with the lowest (12.6 and 2.6 mA·cm^–2^ at 1.5 V, respectively). A similar tendency is observed in terms of mass activity, presenting PtNi/GNPs the best results at various potentials (Figure S4a). These results, along with those related to mass activity, demonstrate the positive effect of adding Ni on both the electrochemical activity and the mass of Pt used. This can be explained according to three factors: first, the oxophilicity of Ni, which is an inherent property that facilitates the dissociation of water molecules and the formation of oxidized species (principally NiOOH), especially in alkaline media and at higher elevated potentials.^44,45^ These compounds enhance the oxidation and desorption of intermediate reaction products derived from the d-glucose electro-oxidation and their subsequent release from neighboring Pt active sites through the bifunctional or Langmuir–Hinshelwood mechanism.^24,46^ The second contribution could be ascribed to the ligand effect. i.e., the variation in the electronic density of Pt due to the alloy formation with Ni, which may affect the adsorption of d-glucose molecules and the subsequent electro-oxidation process.^46,47^ The third contribution involves the blockage of certain Pt sites by Ni atoms, favoring certain reaction pathways without reducing the electrocatalytic activity (ensemble effect).^42^

Additionally, the stability of the electrocatalysts was studied by means of CA tests at 1.1 V vs RHE for three operation cycles, with interspersed OCP steps (Figure 2b). All electrocatalysts exhibited an initial decay in the current density values as a consequence of the poisoning effect caused by the electro-oxidation products at this potential.^42^ These species remain strongly adsorbed on Pt active sites, causing their blockage and impeding the adsorption and electro-oxidation of fresh molecules, hence leading to the decay of the activity. Under the application of the OCP steps, the activity was partially recovered; hence, a similar evolution of the activity was observed over the CA cycles. This suggests that the adsorbed poisoning species are partially removed from the active sites in the OCP steps, with an insignificant contribution of irreversible degradation (such as carbon support corrosion, metal dissolution, or particle sintering). These irreversible effects are more favored at harsh operating conditions of low pH values, high potentials, and high temperatures.^48−52^ Thus, Pt/GNPs and PtNi/GNPs showed a similar general behavior, which was significantly better than that of PtCo/GNPs. However, PtNi/GNPs exhibited again a better average mass activity calculated per operation cycle (Figure S4b), especially at 1.5 V.

##### 3.1.2.3 Electrochemical Experiments in D-Xylose and D-Fructose

The results from d-xylose electro-oxidation are displayed in Figure S5. Similar to d-glucose electro-oxidation, a first peak (Figure S5a) at ∼0.8–0.9 V was observed during the forward scan, associated with the anomeric group dehydrogenation of the d-xylose molecule, followed by another one at ∼1.1 V related to the oxidation of the reaction products and D-xylose molecules.^11^ A higher current density was observed in the second peak compared to the first one, contrary to the results observed in d-glucose electro-oxidation, indicating an apparent enhanced removal of the poisoning species formed compared to that of d-glucose. In this sense, Pt/GNPs showed a higher electrochemical activity at potentials below 1.2 V vs RHE, reaching current density values of 4.5 and 6.5 mA·cm^–2^ at 0.8 and 1.1 V vs RHE, respectively, which are close to those reported in the literature (Table S4). A clear improvement in the electrocatalytic activity was observed at higher potentials (especially with PtNi/GNPs), which could be attributed to the contribution of the oxidized species formed, as previously discussed with glucose. Thus, PtNi/GNPs achieved the highest current density at 1.5 V vs RHE (15.3 mA·cm^–2^), followed by PtCo/GNPs (1.5 times lower) and showing Pt/GNPs the lowest value (almost one-third). Additionally, as observed in the previous study with d-glucose, PtNi/GNPs presented the best results in terms of mass activity (Figure S5b), even at potentials below 1.2 V. According to the results from CA tests at 1.1 V (Figure S5c), Pt/GNPs showed a similar behavior compared to PtNi/GNPs, whereas that of PtCo/GNPs resulted to be substantially poorer in all operating cycles. Similarly, PtNi/GNPs exhibited the highest average mass activity estimated per cycle (Figure S5d), showing higher current density values than those obtained in d-glucose electro-oxidation, especially at higher potentials (e.g., 1.5 V).

Finally, the results derived from d-fructose electro-oxidation are presented in Figure S6. As can be seen in CV measurements (Figure S6a), only a single peak centered at ∼0.7–0.8 V was observed for all electrocatalysts, followed by a quick increase in the electrochemical activity at higher potentials, associated with d-fructose electro-oxidation. However, according to Basu et al.,^13^ this reaction does not undergo a dehydrogenation mechanism on Pt-based electrocatalysts (unlike d-glucose and d-xylose electro-oxidation) which could cause a stronger poisoning effect and thus a lower activity compared to that from these molecules.^13,53^ In this context, similar current density values were achieved at mild potentials with all of the electrocatalysts. Also, a similar activity was observed with Pt/GNPs and PtNi/GNPs at more elevated potentials (10.7 and 11.7 mA·cm^–2^ at 1.5 V, respectively), which resulted to be 2–2.5 times higher than that of PtCo/GNPs (4.7 mA·cm^–2^). These results differed from those previously observed with the other monosaccharides, where PtNi/GNPs showed largely better activity in this potential range. This indicates that despite the previously mentioned positive effects of Ni addition in the electrochemical activity (ligand and ensemble effects and bifunctional mechanism), the probable limited activity provided by this molecule hinders these effects.^13^ However, in terms of mass activity, PtNi/GNPs showed a general better behavior, especially at higher potentials, as revealed in Figure S6b. On the other hand, and according to CA results (Figure S6c), an evident superior stability of PtNi/GNPs was reached with respect to Pt/GNPs, presenting the PtCo/GNPs electrocatalyst the observed lowest activity. This better behavior of PtNi/GNPs was even more remarkable in terms of average mass activity (Figure S6d), in agreement with the trend observed in d-glucose and d-xylose electro-oxidation.

**Figure 3:**
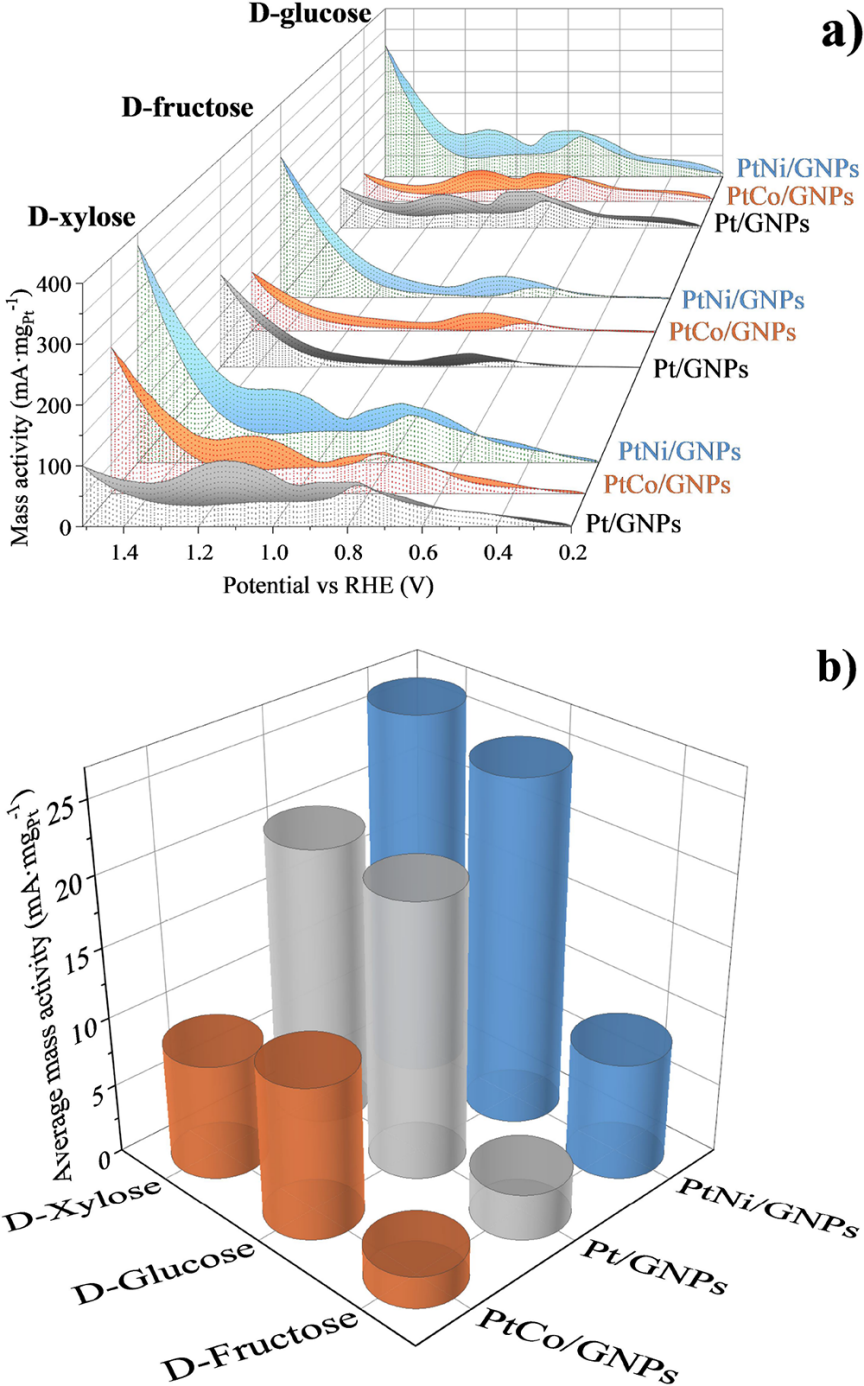
Electrochemical tests with the Pt/GNPs, PtNi/GNPs, and PtCo/GNPs electrocatalysts in 0.1 M monosaccharide + 0.5 M NaOH: (a) CV tests and (b) average mass activity obtained from the different cycles of the CA tests at 1.1 V vs RHE.

All of the results herein discussed are collected in Figure 3. As observed, PtNi/GNPs exhibited the highest mass activity in both CV and CA tests in d-fructose, d-glucose, and d-xylose electro-oxidation, showing this electrocatalyst the best compromise between electrochemical activity and the mass of Pt used. Additionally, the highest activity was achieved in d-xylose electro-oxidation, reaching maximal current density values which were 1.2 and 1.3 times higher (1.5 V) than those in d-glucose and d-fructose electro-oxidation, respectively. For this reason, this molecule was selected to explore the influence of the Pt:Ni mass ratio in the following experiments.

### 3.2 Study of Monometallic (Pt and Ni) and Bimetallic Pt:Ni Electrocatalysts for D-Xylose Electro-Oxidation

The second part of this work involves the study of monometallic (Pt and Ni) and bimetallic PtNi-based electrocatalysts, with Pt:Ni mass ratios of 1:1 and 2:1 used for xylose electro-oxidation. The total metal loading was set at 40 wt % for all cases. Thus, the electrocatalysts were named as Pt, Pt:Ni 2:1, Pt:Ni 1:1, and Ni.

#### 3.2.1 Physicochemical Characterization

**Figure 4:**
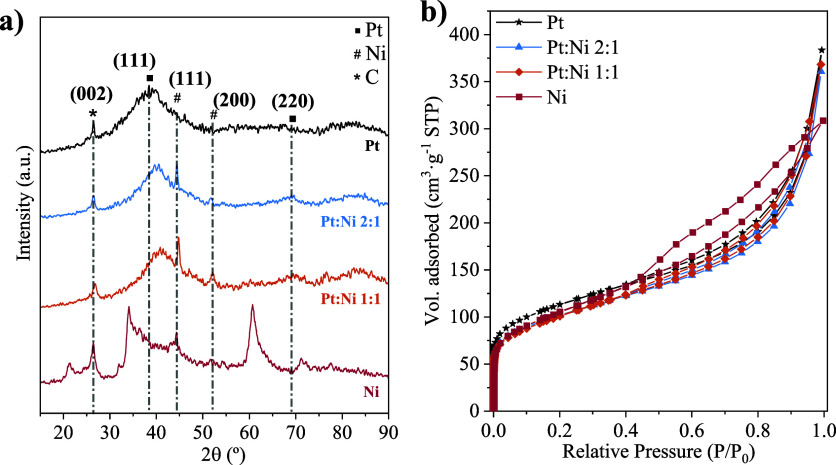
Physicochemical characterization: (a) XRD patterns and (b) N_2_ adsorption–desorption isotherms of Pt, PtNi/GNPs (Pt:Ni 2:1 and 1:1), and Ni electrocatalysts.

Physicochemical characterization results are displayed in Figure 4. According to the XRD patterns (Figure 4a), the Ni electrocatalyst showed two prominent peaks at 2θ = 34.1° and 60.7° corresponding to the planes (012) and (113) of α-Ni(OH)_2_ (JCPDS no. 00-038-0715), respectively, whereas another minor peak was observed at 2θ = 31.9°, which is typical of the (002) reflection of Ni_2_O_3_ (JCPDS no. 00-014-0481).^54^ Peaks centered at 2θ = 52.14° and 44.34° [ascribed to (111) and (200) planes of Ni^44^] presented an expected intensification with increasing Ni content. This indicates the presence of both metallic and oxidized species of Ni in PtNi and Ni electrocatalysts.

Regarding the Pt-based electrocatalysts, a noticeable peak associated with the reflection of the Pt (111) plane was observed in all cases. This peak was shifted toward higher diffraction angles as the Pt:Ni proportion increased due to the formation of a PtNi alloy. The estimated alloy degrees are listed in Table S5, observing an expected increase at rising Ni proportions (i.e., 18.1 and 24% for Pt:Ni 2:1 and Pt:Ni 1:1, respectively). Note that no peaks of planes (012) and (113) of α-Ni(OH)_2_ can be observed in the PtNi catalysts, whereas the peak of the Ni (111) plane (2θ = 44.34°) can be clearly distinguished. Analogously, the sharp peak at 2θ = 60.7° ascribed to the (113) plane of α-Ni(OH)_2_ was not observed either. No diffraction peaks of Pt planes were observed in monometallic Pt catalyst at this angle, and therefore overlapping between Pt and Ni peaks does not occur. This indicates a higher content of the α-Ni(OH)_2_ phase in the Ni electrocatalyst in comparison to Pt:Ni 2:1 and Pt:Ni 1:1.

The isotherms extracted from the N_2_ adsorption–desorption experiments are shown in Figure 4b, while the main textural properties are listed in Table S6. All of the isotherms can be classified as type I–IV, typical of micro- and mesoporous materials. Additionally, an H3-type hysteresis loop was observed in all of the cases, which was more noticeable in the Ni electrocatalyst. The hysteresis loop in isotherms is produced as a consequence of the capillary condensation, which depends on various factors such as temperature and, mainly, pore geometry. Since the temperature of the experiments and the support used were the same in all electrocatalysts, this could be due to a different interaction between the N_2_ molecules and Ni particles with respect to the Pt ones.^33^ Concerning the textural properties, all of the electrocatalysts showed a BET surface area ranging from 358 to 402 m^2^·g^–1^, with the mesopore and micropore volume between 0.477 and 0.540 cm^3^·g^–1^ and 0.108–0.091 cm^3^·g^–1^, respectively.

#### 3.2.2 Electrochemical Experiments in a Three-Electrode Cell

##### 3.2.2.1 CV Tests in 0.5 M NaOH

The electrochemical behavior of the electrocatalysts was determined by CV experiments in 0.5 M NaOH. Figure S7 exhibits the obtained CV curves. As expected, the Pt-based electrocatalysts showed the presence of the peaks ascribed to the (110) and (100)-type sites of Pt in the HUPD region. In the case of the Ni electrocatalyst, a broader peak was observed in this region, mainly associated with the adsorption of OH species on Ni and the subsequent formation of α-Ni(OH)_2_.^55^ In this sense, a clear trend of decreasing current density was observed as the proportion of Ni increased, and Ni atoms entered the Pt crystal structure. This causes an inhibition of the hydrogen adsorption–desorption process due to the poorer electrochemical activity of Ni compared to Pt, as commented above.^56^

Then, the electrochemical activity of the studied PtNi electrocatalysts was explored in the electro-oxidation of d-xylose.

##### 3.2.2.2 Electrochemical Experiments in D-Xylose

**Figure 5:**
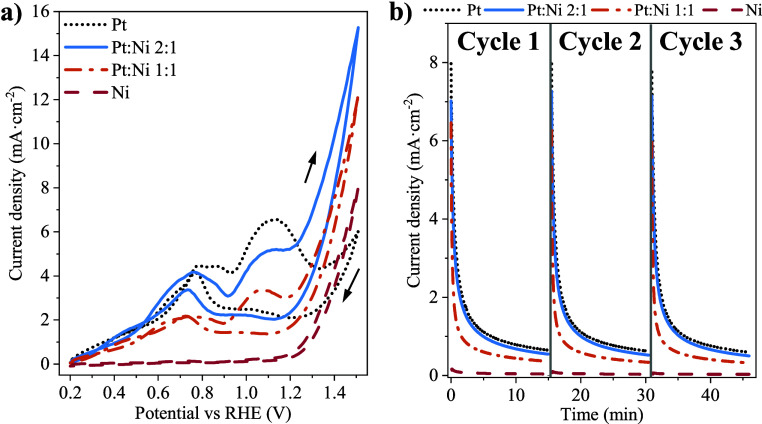
Electrochemical characterization of the mono and PtNi bimetallic electrocatalysts in 0.1 M d-xylose + 0.5 M NaOH at room pressure and temperature: (a) CV experiments at 10 mV·s^–1^ and (b) CA tests at 1.1 V vs RHE.

Figure 5 shows the data obtained in electrochemical experiments in the presence of d-xylose. As expected, a higher electrochemical activity at potentials below 1.2 V was observed as the Pt proportion increased (i.e., at a lower alloy degree), showing no activity with the pure Ni catalyst. However, at higher potentials, the positive effect of Ni addition (with the subsequent formation of NiOOH) was noticeable in such a manner that a higher Ni proportion (i.e., a higher alloy degree) led to a higher activity, showing the Pt electrocatalyst even a poorer activity than that of the Ni one at 1.5 V. In this sense, Pt:Ni 2:1 exhibited higher average mass activity values estimated per cycle (Figure S8a) at the potentials of 0.7 and 1.1 V (95.5 and 127.4 mA·mg_Pt_^–1^). Considering this, a clear positive effect of a higher PtNi alloy degree can be observed in the electrochemical activity at higher potentials. Regarding the CA tests at 1.1 V (Figure 5b), a decrease in the current density values was observed as the Ni ratio increased, showing no activity of the Ni electrocatalyst at this potential. Thus, the Pt:Ni 2:1 and Pt electrocatalysts exhibited a similar behavior, presenting the first one with an improvement in terms of mass activity, as displayed in Figure S8b.

For this reason, the Pt:Ni mass ratio of 2:1 was selected as the optimal in terms of electrochemical activity and the mass of Pt used and was used for a scale-up to an AEM electrolyzer.

### 3.3 Scale-Up to an AEM Electrolyzer

**Figure 6:**
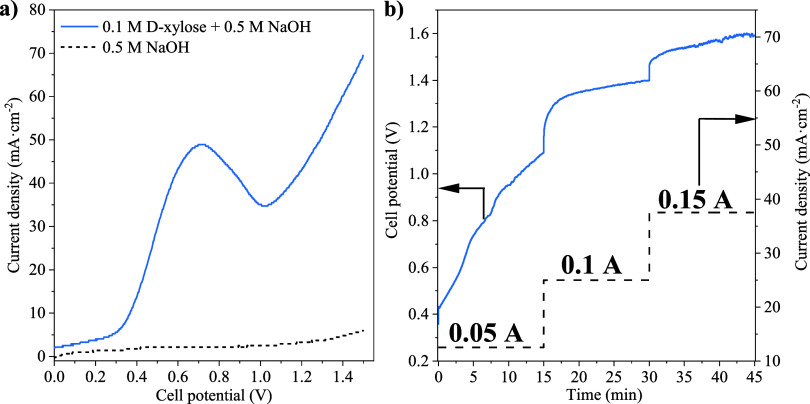
Electrochemical characterization in an AEM electrolyzer with the Pt:Ni 2:1 electrocatalyst in 0.1 M d-xylose +0.5 M NaOH: (a) LSV experiments at a scan rate of 20 mV·s^–1^ and (b) CP tests at various intensity levels. Temperature: 50 °C.

Considering all previous studies, the electrochemical reforming of d-xylose was explored in an AEM electrolyzer using a Pt:Ni 2:1 electrocatalyst as an anode. The obtained results are collected in Figure 6. First, LSV tests (Figure 6a) were performed at a cell potential range from 0 to 1.5 V using 0.5 M NaOH and 0.1 M d-xylose +0.5 M NaOH aqueous solutions as feeding streams for the cathode and anode, respectively. Additionally, 0.5 M NaOH was also fed to the anode for comparison purposes. In good agreement with previous results, a superior electrochemical activity with 0.1 M d-xylose was observed in comparison with the 0.5 M NaOH solution, reaching a current density of 69.6 and mA·cm^–2^ at a cell potential of 1.5 V. In this context, a first increase of the current density, followed by a sudden decrease, was observed when the d-xylose solution was fed, showing a peak centered at 0.7 V. This could be due to a probable poisoning effect caused by the adsorption of electro-oxidation intermediate molecules or reaction products, similar to the results from CV tests in a three-electrode cell. A further increase in the activity was appreciated above a cell potential of 1 V, where the simultaneous oxidation of these intermediate reaction products and fresh d-xylose molecules takes place. The absence of another peak at higher cell potentials implies improved dynamics in the removal of these intermediates, suggesting better performance of the system within this specific cell potential range. In agreement with these results, CP tests (Figure 6b) exhibited a fast increase in the cell potential (49.1 mV·min^–1^) at low current density values (12.5 mA·cm^–2^), showing no evidence of cell potential stabilization. Then, a slower increment of the cell potential at higher current density values was observed, resulting in visible enhanced stability. Thus, an increment of 17.9 and 10.3 mV·min^–1^ was obtained for the current densities of 25 and 37.5 mA·cm^–2^ (0.1 and 0.15 A), respectively. This could be due to the favored oxidation of the adsorbed species responsible for the electrocatalyst deactivation effect given at a cell potential above 1 V, as observed in the LSV results.

**Figure 7:**
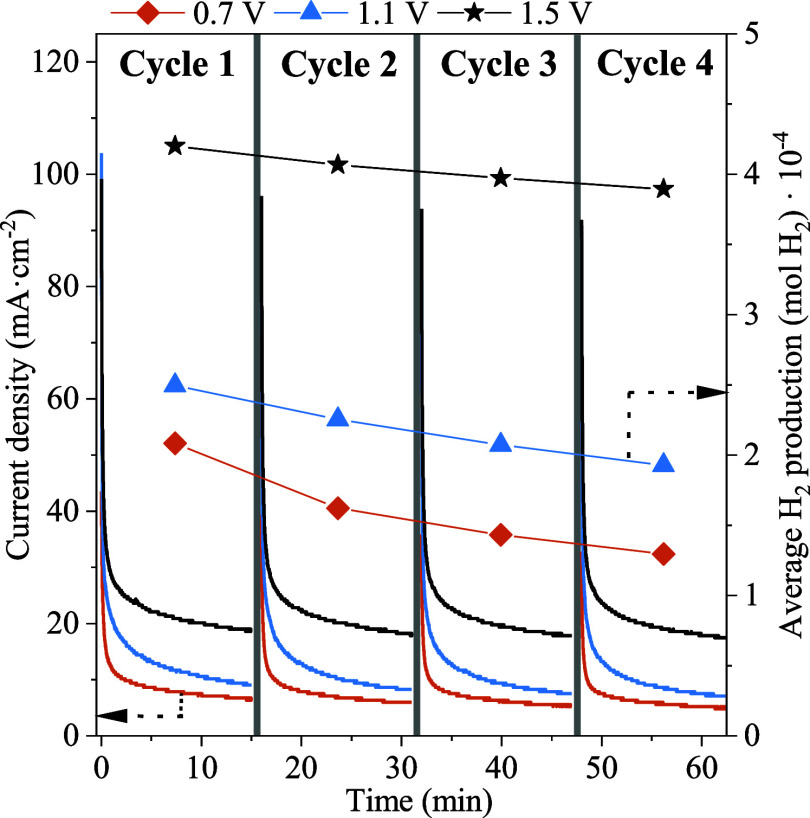
CA tests at various cell potentials in an AEM electrolyzer with the Pt:Ni 2:1 electrocatalyst in 0.1 M d-xylose +0.5 M NaOH. Temperature: 50 °C.

Then, the optimal operating potential of the electrolyzer was determined through CA tests at the applied cell potentials of 0.7, 1.1, and 1.5 V for four cycles of 15 min each. The regeneration method used (named as OCP) consisted of the application of interspersed OCP steps of 1 min between the operating potential cycles. The results obtained are shown in Figure 7. As observed, the Pt active sites were partially released after the OCP steps, showing an operating cycle to start again at higher current density values. Thus, higher current density values (and hence calculated average H_2_ production values) were obtained as the applied potential increased. However, a higher increment in the activity was observed between the CA tests at 1.1 and 1.5 V compared to those at 0.7 and 1.1 V, hence confirming the enhanced removal of reaction intermediates decreasing the deactivation rate induced by the adsorbed electro-oxidation products. However, a deactivation effect was observed in the CA cycles at this cell potential, leading to a progressive decrease in H_2_ production along the cycles.

To mitigate the observed electrocatalyst deactivation effect, particularly at 1.5 V, an alternative regeneration method (named cathodic polarization) was investigated as an alternative for the 1 min OCP steps used in the previous figure. This method consisted of a prior OCP step of 15 s after the operation cycle at 1.5 V, followed by the application of a cell potential of −1.5 V for 30 s. Then, another 15 s OCP step was incorporated before the next operation cycle at 1.5 V. The aim of incorporating the OCP steps was to avoid the extreme difference in the applied cell potential (from 1.5 to −1.5 V). Thus, this regeneration method was studied on the basis that the application of a negative potential could enhance the removal of adsorbed reaction intermediates on Pt active sites more efficiently than when a negative potential (OCP) is applied.

**Figure 8:**
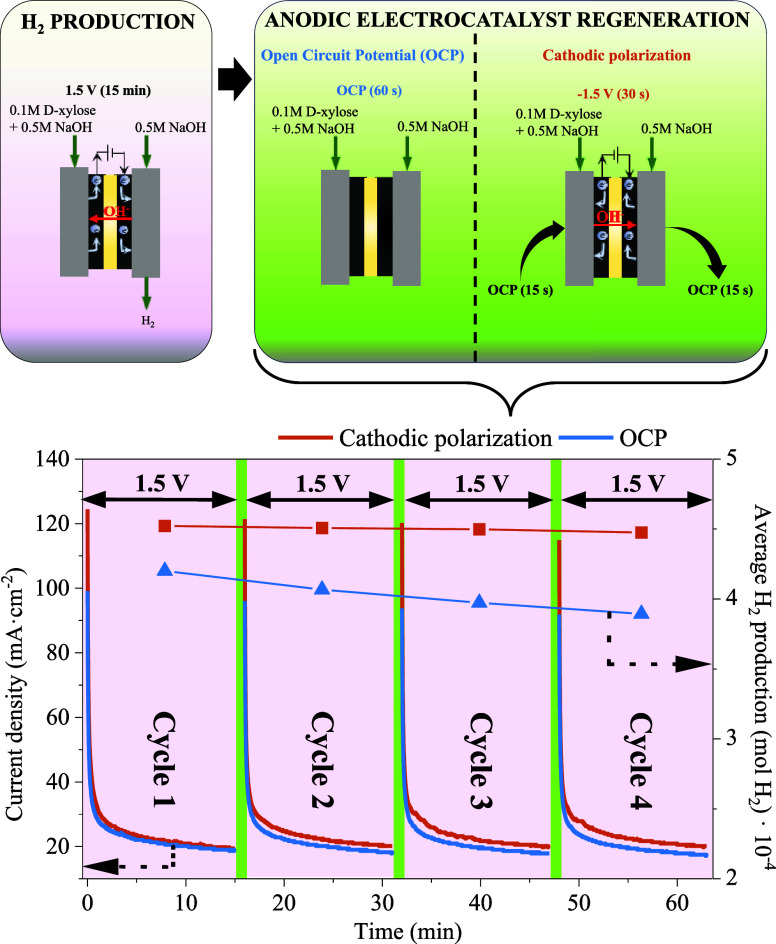
Methodology used for the studied regeneration methods and the results obtained from CA tests.

Therewith, the methodology used and the CA cycles obtained applying both regeneration methods are displayed in Figure 8. A lower decrease in the average current density values can be observed over the operating cycles when the cathodic polarization is used instead of the OCP, with estimated average H_2_ production values practically constant over the operating cycles. Considering this, when using the cathodic polarization, the average H_2_ production resulted 7.7% higher in the first operating cycle in comparison to OCP, whereas for the last (fourth) cycle resulted to be 14.9% higher, hence confirming the improved stability obtained with this method. For this reason, cathodic polarization is proposed as a promising alternative regeneration method for PtNi-based anodic electrocatalysts in view of future technical applications.

**Figure 9:**
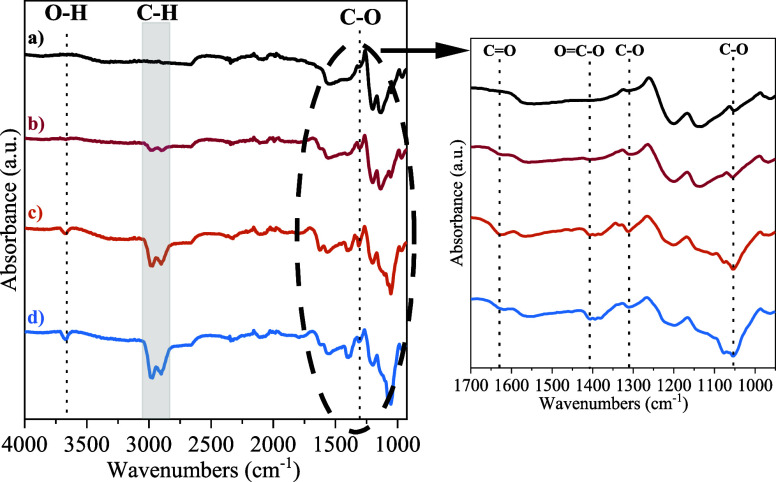
FTIR spectra. (a) Unused electrocatalyst, (b) after immersion in the OCP conditions, (c) after the application of 1.5 V and the OCP cycle, and (d) after the application of 1.5 V and the cathodic polarization cycle.

Finally, in order to better understand the reaction products and the regeneration mechanism, *ex situ* FTIR characterization experiments were conducted on the selected Pt:Ni 2:1 electrocatalyst. For that purpose, the electrocatalyst surface (deposited on a carbon paper GDL) was analyzed under four different cases: (a) fresh catalyst (blank), (b) after 15 min under OCP conditions in 0.1 M d-xylose +0.5 M NaOH, (c) after 15 min under 1.5 V in 0.1 M d-xylose +0.5 M NaOH, and (d) after 15 min of 1.5 V and a 1 min cathodic polarization cycle. FTIR spectra are shown in Figure 9.

Thus, two prominent peaks were principally observed at 2800–3050 cm^–1^ in the cases of (c) and (d), while their presence in (b) was comparatively less pronounced. Notably, these peaks were not detected in the fresh electrocatalyst (case a). These peaks are related to the stretching of C–H bonds from probably d-xylose molecules, which remain adsorbed on the surface of the electrocatalyst once introduced in the monosaccharide solution, even without the application of potential (case b). However, the observed smaller peaks suggest a poorer adsorption of these molecules in the latter case.

Concerning the cases in contact with the d-xylose solution, a small peak centered at 3670 cm^–1^ was observed only after the application of potential (cases b and c), which is ascribed to the stretching vibration of O–H bonds from both the d-xylose molecules and the reaction intermediates adsorbed on the electrocatalyst.^57^ This confirms that the adsorption of d-xylose on the electrocatalyst surface takes place mainly under the application of potential, as commented above. Additionally, the peaks at 1408 and 1054 cm^–1^ related to O=C–O and C–O stretching resulted in higher (c) and (d).^58^ These groups can be ascribed to xylonate groups, which are formed after d-xylose electro-oxidation at a cell potential of 1.5 V.^59^ This suggests that these reaction products remain on the electrocatalyst surface regardless of the regeneration method applied.

Focusing on the differences found between the regeneration methods explored (cases c and d), the peaks at 1630 and 1310 cm^–1^ resulted higher in (c) (after OCP regeneration). These bands are associated with the stretching of C= O and C–O bonds from xylonate groups,^60^ which suggests that these groups are found to a lesser extent after the application of the cathodic polarization (case d). This can be explained as when a negative potential is applied (−1.5 V), the cathodic (reduction) reaction is now given in the Pt:Ni 2:1 electrocatalyst. Thus, the reaction intermediates (xylonate) could be reduced probably into d-xylose, which could be removed more easily from the Pt active sites than xylonate species.^43^ This could lead to enhanced stability during the operating cycles observed in Figure 8. In this sense, cathodic polarization is posed as an excellent method to be considered for electrocatalyst regeneration in future technical applications.

## 4 Conclusions

In this work, Pt, PtNi, and PtCo supported on GNPs electrocatalysts (with a metal loading of 40 wt % and a Pt:M mass ratio of 2:1 in the case of the bimetallic ones) were synthesized by the modified polyol method and studied for the electro-oxidation of d-xylose, d-glucose, and d-fructose. The PtCo/GNPs electrocatalyst showed generally poorer electrochemical activity and stability, probably due to the contribution of various unfavorable factors, such as a lower BET surface area, a broader crystallite size distribution, and a higher alloy degree which reduces the number of available Pt active sites. The PtNi/GNPs electrocatalyst showed the best electrochemical activity and stability in terms of mass activity, reaching in d-xylose a current density value 1.2 and 1.3 times higher (at 1.5 V vs RHE) than in d-glucose and d-fructose, respectively. Thus, various Pt:Ni mass ratios were explored in d-xylose electro-oxidation. An overall improvement of the electrochemical activity was observed as the Pt proportion increased at potentials below 1.2 V. However, at higher potentials, the addition of Ni exhibited a noticeable promotional effect. This is probably due to the formation of Ni-oxidized species (probably in the form of NiOOH), which reduces the electrocatalyst poisoning through a bifunctional mechanism.

Based on these findings, the Pt:Ni mass ratio of 2:1 presented the best balance between electrochemical activity and the mass of Pt used. Consequently, it was used as the anode in an AEM electrolyzer for the electroreforming of d-xylose. In this case, a clear improved stability was observed at higher cell potentials in CP experiments due to the probable enhanced removal of the reaction intermediate species at potentials higher than 1 V, according to LSV tests. In addition, the operating cell potentials of 0.7, 1.1, and 1.5 V were explored in CA assays. Thus, the operating cell potential of 1.5 V exhibited higher activity in CA assays and was selected as the optimal. On the other hand, cathodic polarization regeneration was explored as a method for electrocatalyst regeneration alternative to the synthesis of OCP. Under these regeneration conditions, a superior stability could be observed in the 1.5 V operating cycles, achieving a constant hydrogen production. This was attributed to the removal of xylonate species derived from d-xylose electro-oxidation according to FTIR results.

## Supporting Information Available

Data extracted from XRD patterns; textural properties obtained from N_2_ adsorption-desorption tests; HRTEM images and corresponding particle size distribution of Pt/GNP, PtNi/GNP, and PtCo/GNP electrocatalysts; CV tests of Pt/GNP, PtNi/GNP, and PtCo/GNP electrocatalysts; comparison between the studies concerning alkaline glucose electro-oxidation; electrochemical characterization of Pt/GNP, PtNi/GNP, and PtCo/GNP electrocatalysts; comparison between the studies concerning alkaline xylose electro-oxidation (PDF)
