# Supplementary material for: Boosting the Electrolysis of Monosaccharide-Based Streams in an Anion-Exchange Membrane Cell
Source: Energy Fuels. 2024 May 20;38(11):10038–49. doi: 10.1021/acs.energyfuels.4c00136 (PMC11164063; doi:10.1021/acs.energyfuels.4c00136)
Supplement: Supplementary file 1 — ef4c00136_si_001.pdf [file ef4c00136_si_001.pdf]

Supporting Information for Publication

Boosting the electrolysis of monosaccharide-based streams in an Anion Exchange Membrane cell

J. Serrano-Jiménez [1], A.R. de la Osa [1], P. Sánchez [1], A. Romero [2], A. de Lucas-Consuegra [1*]

[1] Department of Chemical Engineering, School of Chemical Sciences and Technologies, University of Castilla-La Mancha, Avda. Camilo José Cela 12, E-13071, Ciudad Real, Spain.

[2] Department of Chemical Engineering, Higher Technical School of Agronomical Engineers, University of Castilla-La Mancha, Ronda de Calatrava 7, E-13071, Ciudad Real, Spain.

Corresponding author:
* Antonio.Lconsuegra@uclm.es

Table S.1. Data extracted from XRD patterns.

Catalyst	Pt (1 1 1) position (2θ, º)	d-spacing (nm)	lattice constant (nm)	Alloying degree (%)
Pt/GNPs	39.1	0.230	0.399	-
PtNi/GNPs	40.0	0.225	0.390	18.1
PtCo/GNPs	40.7	0.221	0.383	30.7

Table S.2. Textural properties obtained from N_2 adsorption-desorption tests.

Sample	BET Surface Area ($m^2 \cdot g^{-1}$)	Mesopore Volume ($cm^3 \cdot g^{-1}$)	Micropore Volume ($cm^3 \cdot g^{-1}$)	Total pore Volume ($cm^3 \cdot g^{-1}$)
GNPs	739.2 ± 1.5	1.117	0.198	1.315
Pt/GNPs	402.3 ± 1.7	0.540	0.108	0.648
PtNi/GNPs	362.2 ± 1.1	0.522	0.096	0.618
PtCo/GNPs	158.9 ± 0.5	0.349	0.026	0.375

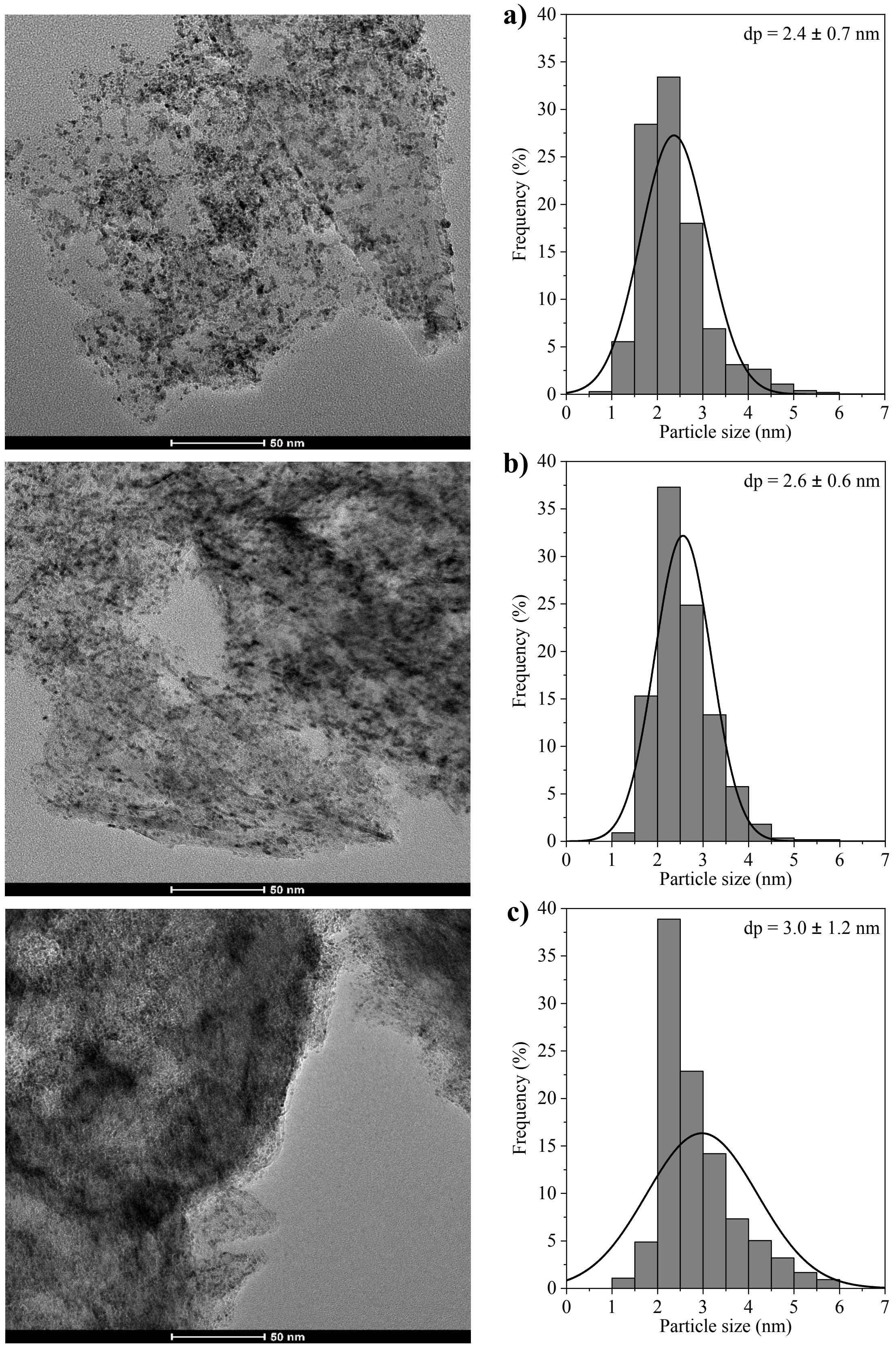

Figure S.1. HRTEM images and corresponding particle size distribution of **a)** Pt/GNPs **b)** PtNi/GNPs and **c)** PtCo/GNPs electrocatalysts.

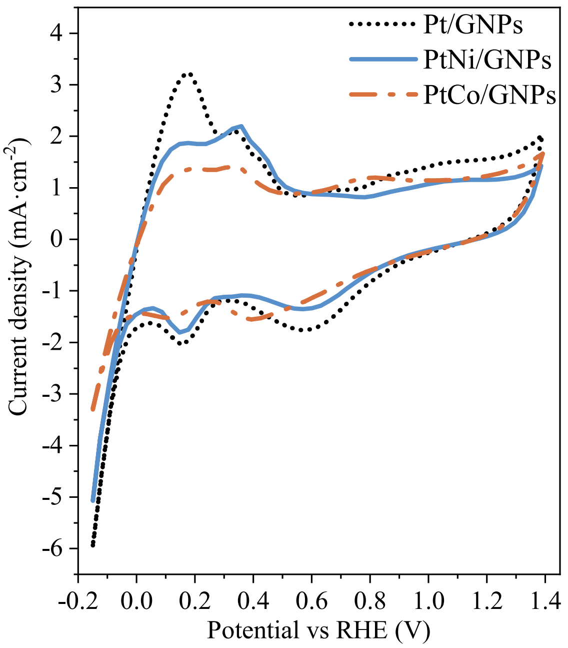

Figure S.2. Cyclic voltammetry tests of Pt/GNPs, PtNi/GNPs and PtCo/GNPs electrocatalysts in 0.5 M NaOH. Conditions: 50 $mV\cdot s^{-1}$, room pressure and temperature.

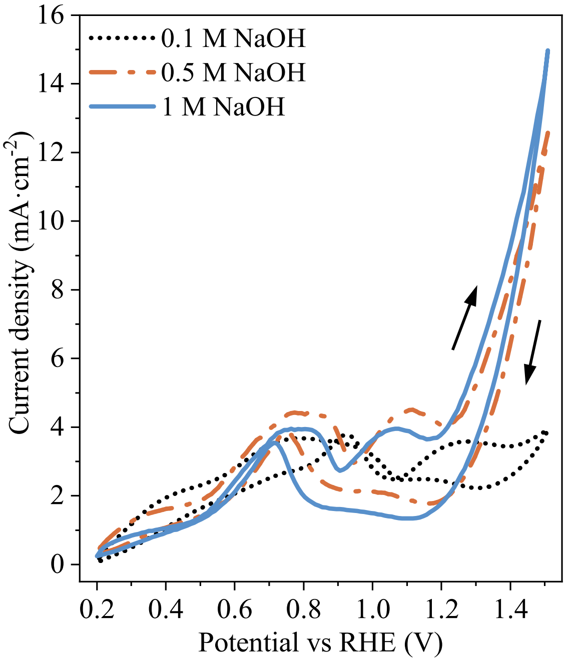

Figure S.3. Cyclic voltammetry tests of **PtNi/GNPs** electrocatalyst using 0.1 M D-glucose solutions and various NaOH concentrations. Conditions: 10 $mV\cdot s^{-1}$, room pressure and temperature.

Table S.3. Comparison between the studies concerning alkaline glucose electro-oxidation.

Solution	Anode	j_{max} (mA·cm^{-2})	Potential (vs RHE)	Scan rate (mV·s^{-1})	Ref.
0.1 M glucose + 0.1 M NaOH	Pd/C	~4.5	0.9	5	[1]
0.1 M glucose + 0.1 M NaOH	Au/C	~4	0.5	5	[1]
0.1 M glucose + 0.1 M NaOH	Pt/C	1.54	0.66	5	[2]
0.1 M glucose + 0.1 M NaOH	Pt-NPs/C	~2.5	0.4	5	[3]
0.04 M glucose + 0.1 M NaOH	Pt electrode	~0.5	~1.1	10	[4]

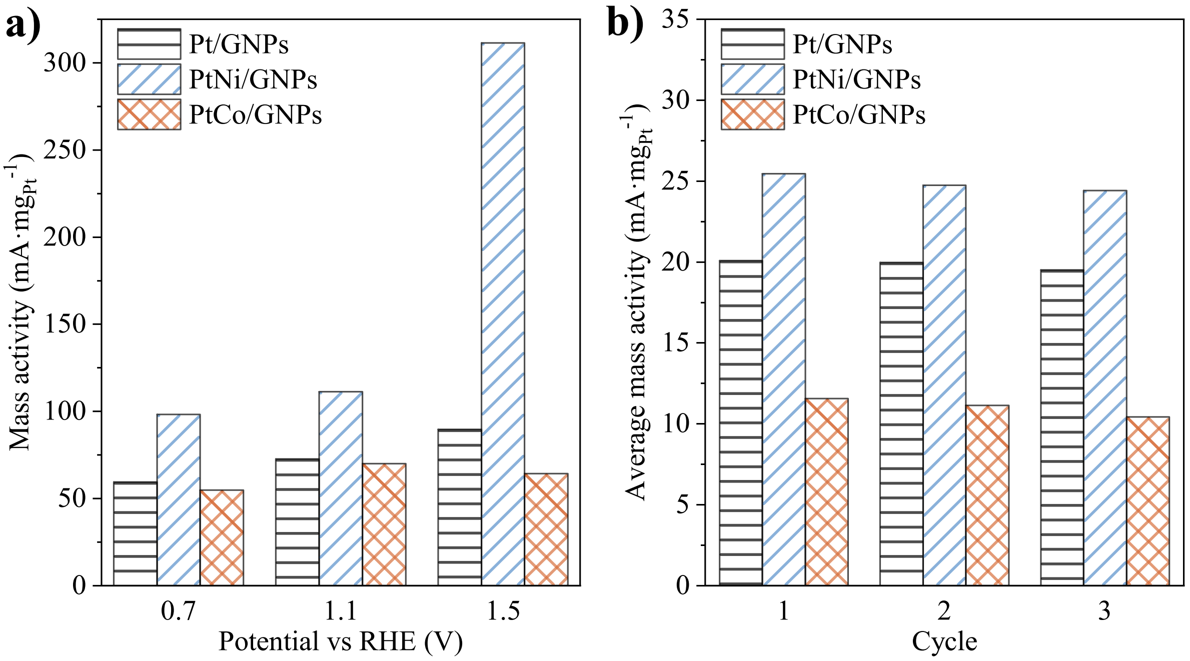

Figure S.4. Electrochemical characterization of the Pt/GNPs, PtNi/GNPs and PtCo/GNPs electrocatalysts in 0.1 M **D-glucose** + 0.5 M NaOH at room pressure and temperature: **a)** Mass activity values obtained from CV at various potentials, and **b)** Average mass activity values calculated from each CA cycle at 1.1 V vs RHE.

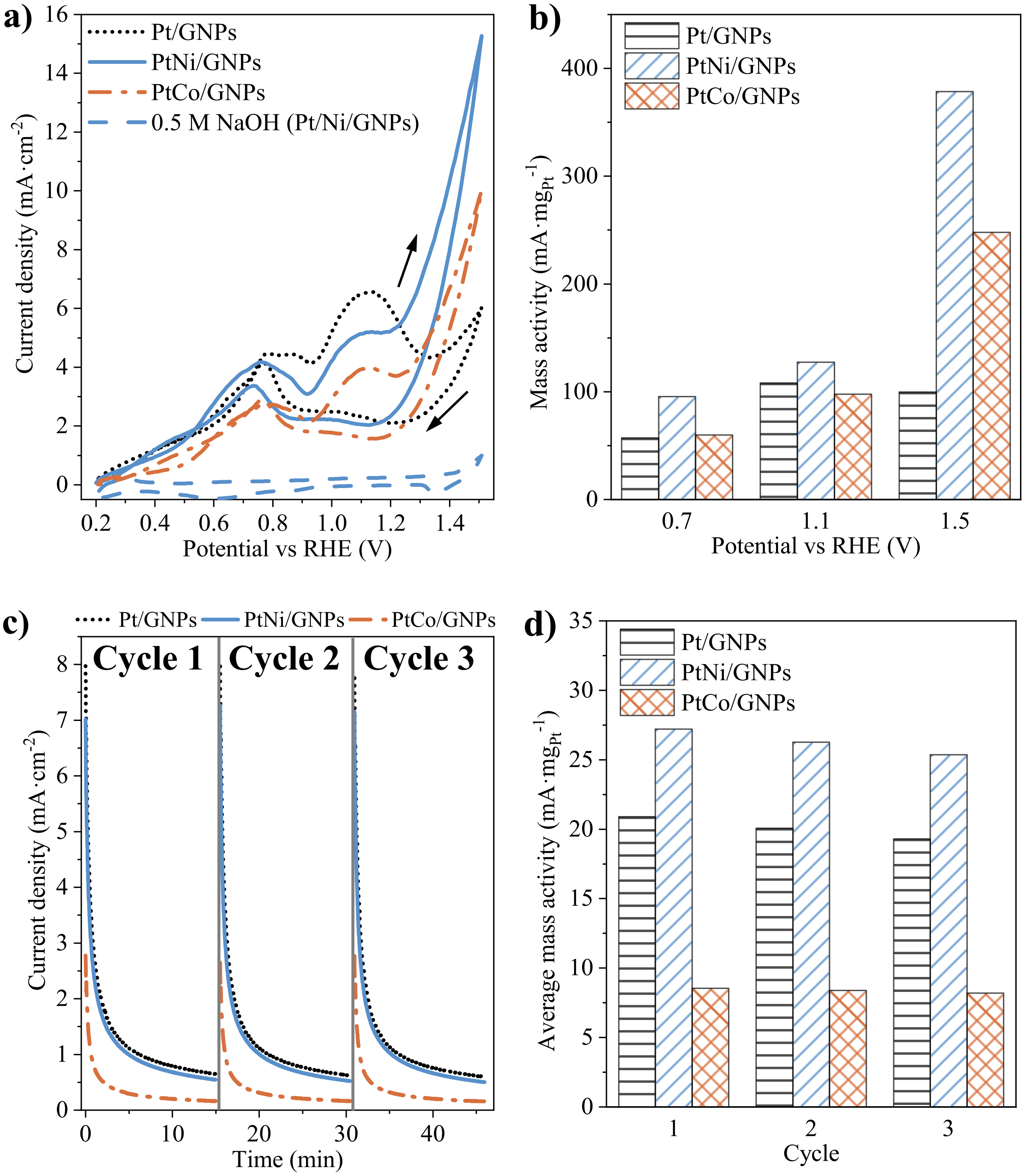

Figure S.5. Electrochemical characterization of the Pt/GNPs, PtNi/GNPs and PtCo/GNPs electrocatalysts in 0.1 M **D-xylose** + 0.5 M NaOH or 0.5 M NaOH at room pressure and temperature: **a)** CV experiments at a scan rate of 10 $mV \cdot s^{-1}$, **b)** Mass activity values obtained from CV at various potentials, **c)** CA tests at 1.1 V vs RHE, and **d)** Average mass activity values calculated from each CA cycle.

Table S.4. Comparison between the studies concerning alkaline xylose electro-oxidation.

Solution	Anode	j_{max} ($mA \cdot cm^{-2}$)	Potential (vs RHE)	Scan rate ($mV \cdot s^{-1}$)	Ref.
0.1 M xylose + 0.1 M NaOH	Pd/C	~4.5	0.9	5	[1]
0.1 M xylose + 0.1 M NaOH	Au/C	~4	1.2	5	[1]
0.1 M xylose + 0.1 M NaOH	Pd_3Au_7/C	~4.7	1.0	5	[2]
0.01 M xylose + 0.1 M NaOH	Pt electrode	0.52	1.07	50	[3]
0.01 M xylose + 0.1 M NaOH	Au electrode	6.56	1.05	50	[4]

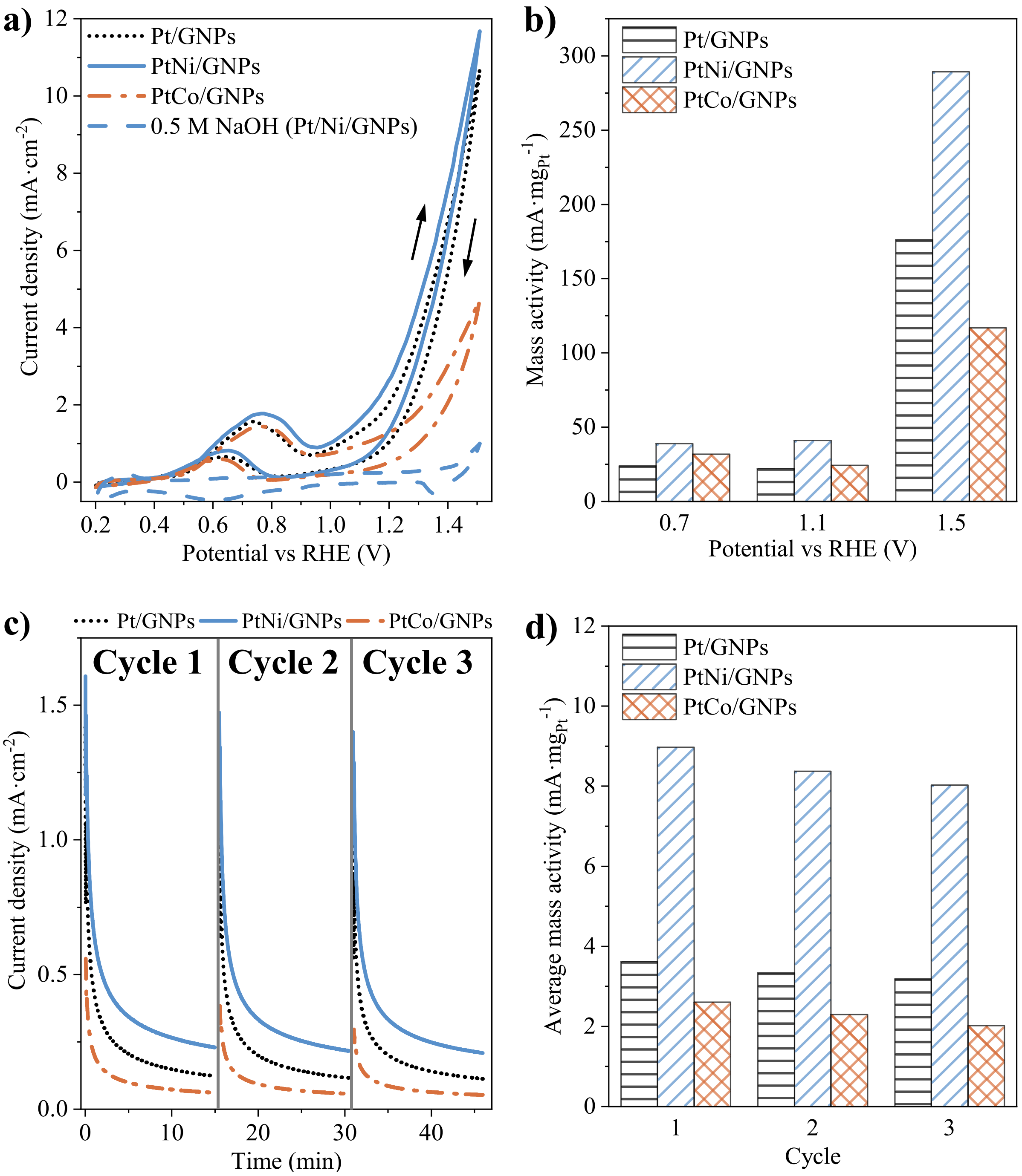

Figure S.6. Electrochemical characterization of Pt/GNPs, PtNi/GNPs and PtCo/GNPs electrocatalysts in 0.1 M **D-fructose** + 0.5 M NaOH or 0.5 M NaOH at room pressure and temperature: **a)** CA experiments at a scan rate of 10 $mV·s^{-1}$, **b)** Mass activity obtained from CV at various potentials. **c)** CA tests at 1.1 V, and **d)** Average mass activity calculated from each CA cycle.

Table S.5. Data extracted from XRD patterns.

Catalyst	Pt (1 1 1) position (2θ, º)	d-spacing (nm)	lattice constant (nm)	Alloying degree (%)
Pt	39.1	0.230	0.399	-
Pt:Ni 2:1	40.0	0.225	0.390	18.1
Pt:Ni 1:1	40.3	0.224	0.388	24.0

Table S.6. Textural properties obtained from N_2 adsorption-desorption tests.

Sample	BET Surface Area ($m^2 \cdot g^{-1}$)	Mesopore Volume ($cm^3 \cdot g^{-1}$)	Micropore Volume ($cm^3 \cdot g^{-1}$)	Total pore Volume ($cm^3 \cdot g^{-1}$)
Pt	402.3 ± 1.7	0.540	0.108	0.648
Pt:Ni 2:1	362.2 ± 1.1	0.522	0.096	0.618
Pt:Ni 1:1	358.6 ± 1.0	0.542	0.094	0.636
Ni	375.5 ± 0.6	0.477	0.091	0.568

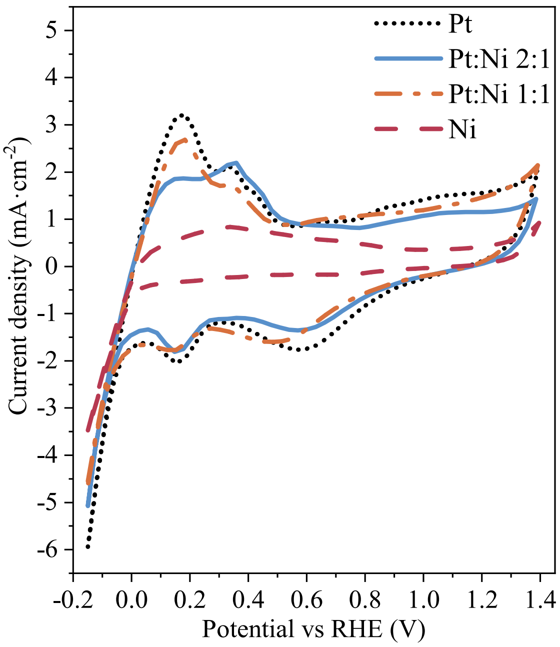

Figure S.7. Cyclic voltammetry tests of mono and PtNi bimetallic electrocatalysts in 0.5 M NaOH. Conditions: 50 $mV \cdot s^{-1}$, room pressure and temperature.

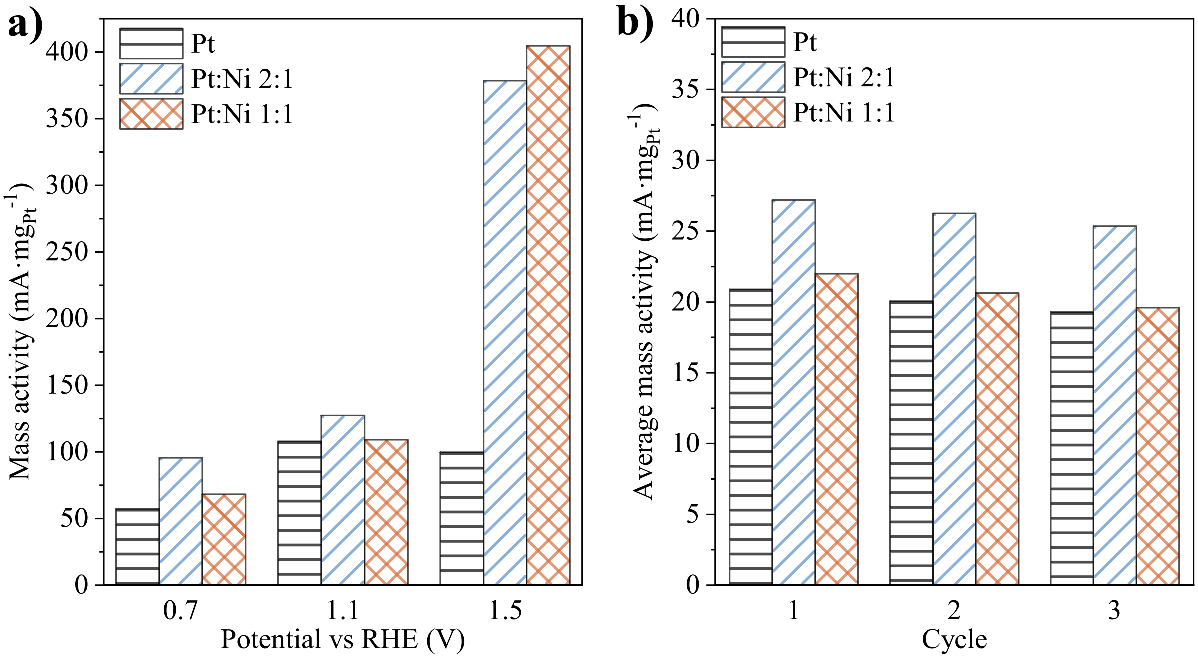

Figure S.8. Electrochemical characterization of the mono and PtNi bimetallic electrocatalysts in 0.1 M **D-xylose** + 0.5 M NaOH at room pressure and temperature: **a)** Mass activity values obtained from CV at various potentials, and **b)** Average mass activity values calculated from each CA cycle at 1.1 V vs RHE.

References

[1] T. Rafaïdeen, S. Baranton, C. Coutanceau. Highly efficient and selective electrooxidation of glucose and xylose in alkaline medium at carbon supported alloyed PdAu nanocatalysts. Applied Catalysis B. 243 (2019) 641–656. https://doi.org/10.1016/J.APCATB.2018.11.006.

[2] N. Neha, B.S.R. Kouamé, T. Rafaïdeen, S. Baranton, C. Coutanceau. Remarkably Efficient Carbon-Supported Nanostructured Platinum-Bismuth Catalysts for the Selective Electrooxidation of Glucose and Methyl-Glucoside. Electrocatalysis 12 (2021) 1–14. https://doi.org/10.1007/s12678-020-00586-y.

[3] N. Neha, T. Rafaïdeen, T. Faverge, F. Maillard, M. Chatenet, C. Coutanceau. Revisited mechanisms for glucose electrooxidation at platinum and gold nanoparticles. Electrocatalysis 14 (2022) 121-130. https://doi.org/10.1007/s12678-022-00774-y.

[4] G. Moggia, T. Kenis, N. Daems, T. Breugelmans. Electrochemical oxidation of D-glucose in alkaline medium: impact of oxidation potential and chemical side reactions on the selectivity to D-gluconic and D-glucaric acid. ChemElectroChem 7 (2020) 86–95. https://doi.org/https://doi.org/10.1002/celc.201901592.
